# Role of ADAMTS-5 in Aortic Dilatation and Extracellular Matrix Remodeling

**DOI:** 10.1161/ATVBAHA.117.310562

**Published:** 2018-04-05

**Authors:** Marika Fava, Javier Barallobre-Barreiro, Ursula Mayr, Ruifang Lu, Athanasios Didangelos, Ferheen Baig, Marc Lynch, Norman Catibog, Abhishek Joshi, Temo Barwari, Xiaoke Yin, Marjan Jahangiri, Manuel Mayr

**Affiliations:** 1 From the King’s British Heart Foundation Centre, King’s College London, United Kingdom (M.F., J.B.-B., U.M., R.L., A.D., F.B., M.L., N.C., A.J., T.B., X.Y., M.M.); 2 St George’s University of London, NHS Trust, United Kingdom (M.F., M.J.); 3 Cardiovascular Institute, Cardiovascular Research Center, Icahn School of Medicine at Mount Sinai, New York (M.F., M.M.).

**Keywords:** aneurysm, aorta, extracellular matrix, proteases, proteomics

## Abstract

**Objective—:**

Thoracic aortic aneurysm (TAA), a degenerative disease of the aortic wall, is accompanied by changes in the structure and composition of the aortic ECM (extracellular matrix). The ADAMTS (a disintegrin and metalloproteinase with thrombospondin motifs) family of proteases has recently been implicated in TAA formation. This study aimed to investigate the contribution of ADAMTS-5 to TAA development.

**Approach and Results—:**

A model of aortic dilatation by AngII (angiotensin II) infusion was adopted in mice lacking the catalytic domain of ADAMTS-5 (Adamts5^Δcat^). Adamts5^Δcat^ mice showed an attenuated rise in blood pressure while displaying increased dilatation of the ascending aorta (AsAo). Interestingly, a proteomic comparison of the aortic ECM from AngII-treated wild-type and Adamts5^Δcat^ mice revealed versican as the most upregulated ECM protein in Adamts5^Δcat^ mice. This was accompanied by a marked reduction of ADAMTS-specific versican cleavage products (versikine) and a decrease of LRP1 (low-density lipoprotein-related protein 1). Silencing *LRP1* expression in human aortic smooth muscle cells reduced the expression of *ADAMTS5*, attenuated the generation of versikine, but increased soluble ADAMTS-1. A similar increase in ADAMTS-1 was observed in aortas of AngII-treated Adamts5^Δcat^ mice but was not sufficient to maintain versican processing and prevent aortic dilatation.

**Conclusions—:**

Our results support the emerging role of ADAMTS proteases in TAA. ADAMTS-5 rather than ADAMTS-1 is the key protease for versican regulation in murine aortas. Further studies are needed to define the ECM substrates of the different ADAMTS proteases and their contribution to TAA formation.

## Abstract 2

Supplemental Digital Content is available in the text.

ECM (extracellular matrix) degradation and remodeling is a hallmark of aortic aneurysm formation. The precise mechanisms behind the degradation of ECM components and subsequent dissection of the vessel wall are not completely elucidated. Histologically, diseased aortas present with variable degrees of ECM degeneration within the aortic medial layer, including cystic medial degeneration, elastic fiber fragmentation, and myxomatous degeneration. At the molecular level, aortic degeneration has been associated with vascular smooth muscle cell (SMC) apoptosis^1^ and inflammation.^2^ Factors that contribute to aortic wall degeneration both in humans and in animal models include AngII (angiotensin II),^3^ TGF-β (transforming growth factor-β),^4^ and MMPs (matrix metalloproteinases).^5^ The role of MMPs in the alteration of aortic ECM architecture has been extensively studied in the context of aortic aneurysm development.^5,6^ More recently, a family of metalloproteinases known as ADAMTSs (a disintegrin and metalloproteinase with thrombospondin motifs) has been explored in vascular ECM turnover. In particular, ADAMTS-1, -4, and -5 activities have been implicated in thoracic aortic aneurysm (TAA) formation.^3,7,8^

**See accompanying editorial on page 1425**

ADAMTS-1, -4, and -5 are the main enzymes responsible for large aggregating proteoglycan cleavage.^9,10^ We have previously demonstrated that ADAMTS-5 is reduced in aortas of apolipoprotein E–null mice and that ADAMTS-5 activity affects proteoglycan-mediated lipoprotein retention.^11^ We have also shown that on stent-induced vascular injury, a reduction in ADAMTS-1 and ADAMTS-5 contributes to an accumulation of large aggregating proteoglycans, notably aggrecan and versican.^12^ Similarly, the absence of ADAMTS-5 leads to developmental defects including myxomatous valve malformation. Mice lacking ADAMTS-5 activity cannot degrade versican during the remodeling of the valve cushion resulting in enlarged pulmonary and aortic valve cusps.^13^ LRP1 (low-density lipoprotein-related protein 1), a widely expressed receptor, is known to mediate ADAMTS-5 clearance by promoting its endocytosis. Interestingly, LRP1 has been implicated in aneurysm formation; LRP1 deletion in SMCs profoundly augmented aneurysm formation in the ascending aorta (AsAo) induced by AngII.^14^

In the present article, we aim to characterize regional differences in ADAMTS expression in murine aortas and to explore aortic ECM changes in mice with loss of ADAMTS-5 activity. Because the ECM is an intricate protein network and alterations in proteolytic activity will induce secondary remodeling processes, we used our established proteomics approach for studying the vascular ECM.^15^

## Materials and Methods

An expanded Materials and Methods section is available in the online-only Data Supplement. The data that support the findings of this study are available from the corresponding author on reasonable request. The proteomics data, however, are deposited to the ProteomeXchange Consortium via the PRIDE partner repository with the dataset identifier PXD009410 and 10.6019/PXD009410.

### Animal Experiments

All animal procedures were performed by authorized researchers in the Cardiovascular Division, King’s College London. Housing and animal care was in accordance with the UK Animals (Scientific Procedures) Act 1986. Genotyping of Adamts5^Δcat^ and control (Adamts5^+/+^) mice was performed as previously published.^11^ For AngII infusion, osmotic mini-pumps (Alzet, model 1004) containing AngII (1.44 mg g^−1^ d^−1^ dissolved in saline) were implanted in 10- to 12-week-old male Adamts5^Δcat^ and Adamts5^+/+^ mice derived from littermates of heterozygous breeders (JAX stock no. 005771, B6.129P2-*Adamts5*^tm1Dgen^/J). Animals were euthanized after 4 weeks, and the entire aorta was excised and immediately washed in sterile phosphate-buffered saline. The entire aorta was snap-frozen at −80°C for subsequent proteomic analysis. For gene expression analysis, aortic tissue was divided into anatomically defined regions using a scalpel under a dissecting microscope, followed by immediate storage at −80°C. Aortic diameter was monitored using ultrasound at day 0 (baseline) and after 27 days of AngII treatment using Vevo software version 1.7.

### Mouse Echocardiography

Animals were anesthetized using 5% isoflurane mixed with 1 L/min of 100% oxygen for 45 seconds to 1 minute. Mice were then placed in a supine position on a heating pad with embedded ECG registration. On adequate induction, 1% to 1.5% isoflurane mixed with 1 L/min 100% oxygen was used to maintain a steady state of sedation throughout the procedure. A rectal probe was inserted to continuously monitor the body temperature. Electrode gel was applied to the 4 paws, which were taped to the ECG electrodes. Two-dimensional echocardiographic images of cardiovascular anatomy were obtained by a single operator. Standard and modified parasternal long-axis, suprasternal, longitudinal abdominal, and transverse abdominal views were obtained using Visual Sonics Vevo 2100. Aortic root dimensions (aortic annulus, sinuses of Valsalva, and sinotubular junction) were measured in parasternal long-axis view. AsAo dimensions were measured during systole and diastole in suprasternal view where possible and modified parasternal long-axis view if suprasternal views were considered inadequate. Measurements were taken from leading edge-to-leading edge. Abdominal aorta measurements were made in the transverse abdominal view. All measurements were performed offline (Vevo software version 1.7) by 2 consensus interpreters.

### Blood Pressure Monitoring

The blood pressure was directly measured via an implantable radiotelemetry device. An average of 200 values were acquired every 5 minutes for 18 to 20 hours. Blood pressure was monitored using telemetry probes 2 days before mini-pump implantation and at days 5 and 27 of AngII infusion.

### ECM Protein Extraction From Murine Aortas

Aortic samples were partially thawed and diced into smaller pieces to aid the removal of plasma contaminants and for the effective extraction of ECM proteins.^16,17^ ECM protein enrichment of aortic samples was performed using an adaptation of our previously published 3-step extraction method, involving sequential incubation with 0.5 mol/L NaCl for 2 hours, 0.08% sodium dodecyl sulfate (SDS) for 2 hours, and a final incubation with 4 mol/L guanidine hydrochloride (GuHCl) for 48 hours. After precipitation of GuHCl extracts, protein samples were enzymatically deglycosylated and subjected to in-solution trypsin digestion. Details are given in Online Methods in the online-only Data Supplement.

### Proteomics Analysis of ECM Extracts

After deglycosylation (protocol details are provided in the Online Methods in the online-only Data Supplement), digested peptides were separated on a nanoflow liquid chromatography system, and GuHCl extracts were injected for liquid chromatography-tandem mass spectrometry analysis into a Q Exactive HF Hybrid Quadrupole-Orbitrap mass spectrometer (Thermo Fisher Scientific) for discovery proteomics. Acquisition was performed as previously described.^15^ For targeted MS analysis, a precursor isolation list was created for proteotypic peptides of mouse versican and GuHCl extracts were analyzed via parallel reaction monitoring mode on a Q Exactive HF Hybrid Quadrupole-Orbitrap mass spectrometer. Details are given in Online Methods in the online-only Data Supplement.

### Total Protein Extraction From Murine Aortas

Mouse aortic samples were homogenized in the presence of 300 μL of tissue lysis buffer (0.152 g of Tris, 0.33 g of NaCl, 0.038 g of EGTA, 0.073 g of EDTA, 500 μL of Triton X-100, and 250 μL of 20% SDS) at pH 7.4. Protein concentration was measured using the Pierce BCA Protein Assay Kit, and Western blots were performed following the protocol in the Online Methods in the online-only Data Supplement.

### Cell Culture of Human Arterial SMCs

Samples of internal mammary artery were collected from patients undergoing bypass graft surgery at Leeds General Infirmary, conforming with the principles outlined in the Declaration of Helsinki. Arterial SMCs were extracted and expanded using an explant technique.^18^ SMCs were seeded into 6-well tissue culture plates. Twenty-four hours after seeding, cells were cultured in serum-free DMEM for 1 hour. Ten nanogram per milliliter of recombinant human TGF-β1 (R&D systems) was diluted in serum-free DMEM (1195-045; Gibco), and cells were treated for 24 hours. Changes in versican expression after stimulation were measured by real-time quantitative polymerase chain reaction (PCR) using a TaqMan assay for the corresponding human target. The Major Resources Table is given in the online-only Data Supplement.

### Quantitative Real-Time PCR

RNA extraction was performed using the miRNeasy Mini Kit (Qiagen) following the manufacturers’ protocol. RNA concentration (absorbance at 260 nm) and purity (260/280 nm ratio) were measured in 1 μL of eluted RNA using spectrophotometry (NanoDrop ND-1000; Thermo Scientific). RNA integrity was evaluated using the Agilent Bioanalyzer System and considered acceptable if the RNA integrity number was >7. The RNA was then reverse-transcribed using random hexamers with SuperScript VILO MasterMix (Invitrogen) according to manufacturers’ protocol, with sample preparation being performed on a StarChill PCR rack to maintain low temperature. The reverse transcription product was diluted 1:15 to 1:25 using RNase-free water. TaqMan hydrolysis assays were used for quantitative PCR analysis. Data were analyzed using ViiA 7 software (Applied Biosystems). A minimum of 2 reference genes was used throughout the study, and stability of RNA isolation, reverse transcription, and quantitative PCR was determined by variability of and correlation between reference genes. Analysis of stability was also performed using geNorm, a commonly used algorithm for validation of reference gene stability based on the comparative cycle-to-threshold method.^19^ Relative amounts of the targets were calculated using the 2^−ΔΔCq^ method,^20^ with statistical analysis performed on ΔCq values.

### Immunofluorescence Staining in Murine AsAo

Murine aortas were fixed for 24 hours in 4% formaldehyde at 4°C. In brief, 3-μm sections were deparaffinized in xylene and rehydrated in graded ethanol. The slides were unmasked using hot sodium citrate buffer (pH 6.0), then permeabilized for 20 minutes in 0.1% Triton X-100. Sections were blocked with 10% serum at room temperature, before incubation with primary antibodies against LRP1 and α–smooth muscle actin or species-matched isotopes overnight at 4°C. After washes in PBS with 0.1% Tween 20, sections were incubated for 1 hour at room temperature with the secondary antibody. Nuclei were stained using DAPI for 10 minutes. Sections were visualized under a Nikon spinning disk confocal microscope, and images were acquired using NIS-elements 4.0 software. Scale bars are included in the images as indicated in the figure legends.

### Transfection Experiments in Human Aortic SMCs

Human aortic SMCs were purchased from Lonza and cultured in SMC medium (cc-3183; Lonza) with 5% fetal bovine serum (Lonza). SMCs were seeded into 6-well tissue culture plates, and 24 hours after seeding, cells were transfected with 50 nmol/L of *LRP1* siRNA (Dharmacon, M-004721-01-0005, targeted sequences: GCAAGAAGCCGGAGCAUGA, GAACAAACACACUGGCUAA, GCUAUGAGUUUAAGAAGUU, GCGCAUCGAUCUUCACAAA) using lipofectamine 2000 (Thermo Fisher Scientific) according to the manufacturer’s instructions. Nontargeting siRNA served as control (Dharmacon, D-001206-14-05, targeted sequences: UAAGGCUAUGAAGAGAUAC, AUGUAUUGGCCUGUAUUAG, AUGAACGUGAAUUGCUCAA, UGGUUUACAUGUCGACUAA). Cells were incubated for 6 hours in Opti-MEM (11058-021; Gibco) with lipofectamine and siRNAs. After removal of transfection medium, SMCs were incubated with SMC medium with 5% fetal bovine serum for 48 hours. Then, cells were cultured in serum-free SMC medium for 24 hours. Medium was collected for protein measurements. Cells were harvested for quantitative PCR and Western blot analyses. Major Resources Table is given in the online-only Data Supplement. Four milliliters of conditioned media were concentrated using 0.5 mL columns with 3 kDa filter (Amicon Ultra Centrifugal Filter Device; 3 KD). Five hundred milliliters of samples were added each time and centrifuged at 14 000*g* for 40 minutes at 4°C until the residual volume was around 30 μL. Samples were then washed twice using 500 μL of 0.1% SDS each time. Protein concentrates were then used for Western blots. After conditioned medium collection, cell layers were washed 3× with cold phosphate-buffered saline to remove the residual medium. Cells were scraped off in cold phosphate-buffered saline and centrifugated for 1 minute at 15 000*g*. One hundred milliliters of 1× lysis buffer with 1:100 proteinase inhibitors and 25 mmol/L of EDTA were added to cell pellets. After resuspension, lysed cell pellets were vortexed and incubated for 20 minutes on ice. Protein concentration was measured using Pierce BCA Protein Assay Kit before Western blot analyses.

### Immunofluorescence Staining in Human Aortic Specimen

Human aortic tissues were collected from patients undergoing AsAo replacement surgery at St. George’s Hospital according to the local Research Ethics Committee (London, REC No. 08/H0803/257). Aortic tissues were fixed in 10% formalin and then embedded with paraffin. For antigen retrieval, chondroitinase ABC was applied on 5 μm tissue sections for 2 hours at 37°C. After blocking with 10% fetal bovine serum for 1 hour, sections were coincubated with primary antibodies against full-length versican and versican neoepitope DPEAAE^441^ overnight at 4°C. Species-matched isotype IgGs were used as negative controls. After washes in PBS with 0.1% Tween 20, the appropriate secondary antibody was used for 1 hour at room temperature. Nuclei were stained with DAPI (1:1000 dilution) for 10 minutes. The images were visualized with an inverted Nikon Spinning disc confocal unit and acquired using NIS-elements 4.0 software.

### Statistical Analysis

GraphPad Prism software 7 was used for the analysis. For each experiment, Shapiro–Wilk normality test was applied to test the distribution of the data. If the data were not normally distributed, a nonparametric test was used. The appropriate tests and post hoc analysis were chosen according to the data distribution as specified in each figure legend. Data are represented as mean±SD. For proteomics, statistical analysis was not performed if a protein was undetectable in the majority of samples from both groups compared. This is denoted as not applicable. Data from discovery proteomics are presented as normalized total ion current. Data obtained from targeted proteomics are presented as peak area and adjusted according to the total ion intensity for each sample. The aortic diameters are represented as box-and-whisker plots, with 75th and 25th percentiles; bars represent maximum and minimum values. Differences were assessed using a 2-way ANOVA and Šidák post hoc test. For all statistical analysis, *P*<0.05 was deemed significant.

## Results

### ADAMTS Expression in the Murine Aorta

**Figure 1.**
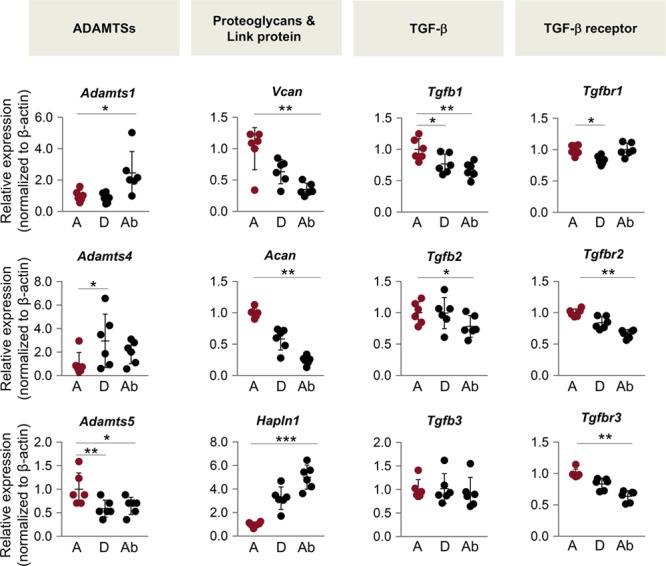
Gene expression along the murine aorta. Relative gene expression of *Adamts1* (a disintegrin and metalloproteinase with thrombospondin motifs 1), *Adamts4* and *Adamts5*, versican (*Vcan*), aggrecan (*Acan*) and hyaluronan and proteoglycan link protein 1 (*Hapln1*), and *Tgfb1* (transforming growth factor-β1), *Tgfb2* and *Tgfb3* and *Tgbr1* (transforming growth factor-β receptor type 1), *Tgbr2* and *Tgbr3* in the aortic arch (A), descending (D) and abdominal (Ab) aorta (n=6). β-actin was used as reference gene. Gene expression levels in the arch served as reference. Values are given as mean±SD; *P<0.05, **P<0.01, ***P<0.001 by Friedman test with Dunn post hoc comparison.

Transcript levels of *Adamts5* were higher in the aortic arch compared with the other aortic regions, along with transcript levels of the large aggregating proteoglycans versican (*Vcan*) and aggrecan (*Acan*), 2 ADAMTS-5 substrates, which were most highly expressed in the arch. Expression of *Tgfb1* and *Tgfb2* and the receptors *Tgfbr2* and *Tgfbr3* were also higher in the arch compared with the abdominal aorta. In contrast, *Adamts1* and *Adamts4* and hyaluronan and proteoglycan link protein 1 (*Hapln1*) showed lower transcript levels in the arch compared with other regions of the murine aorta (Figure 1).

### Aortic Dilatation in Adamts5^Δcat^ Mice

**Figure 2.**
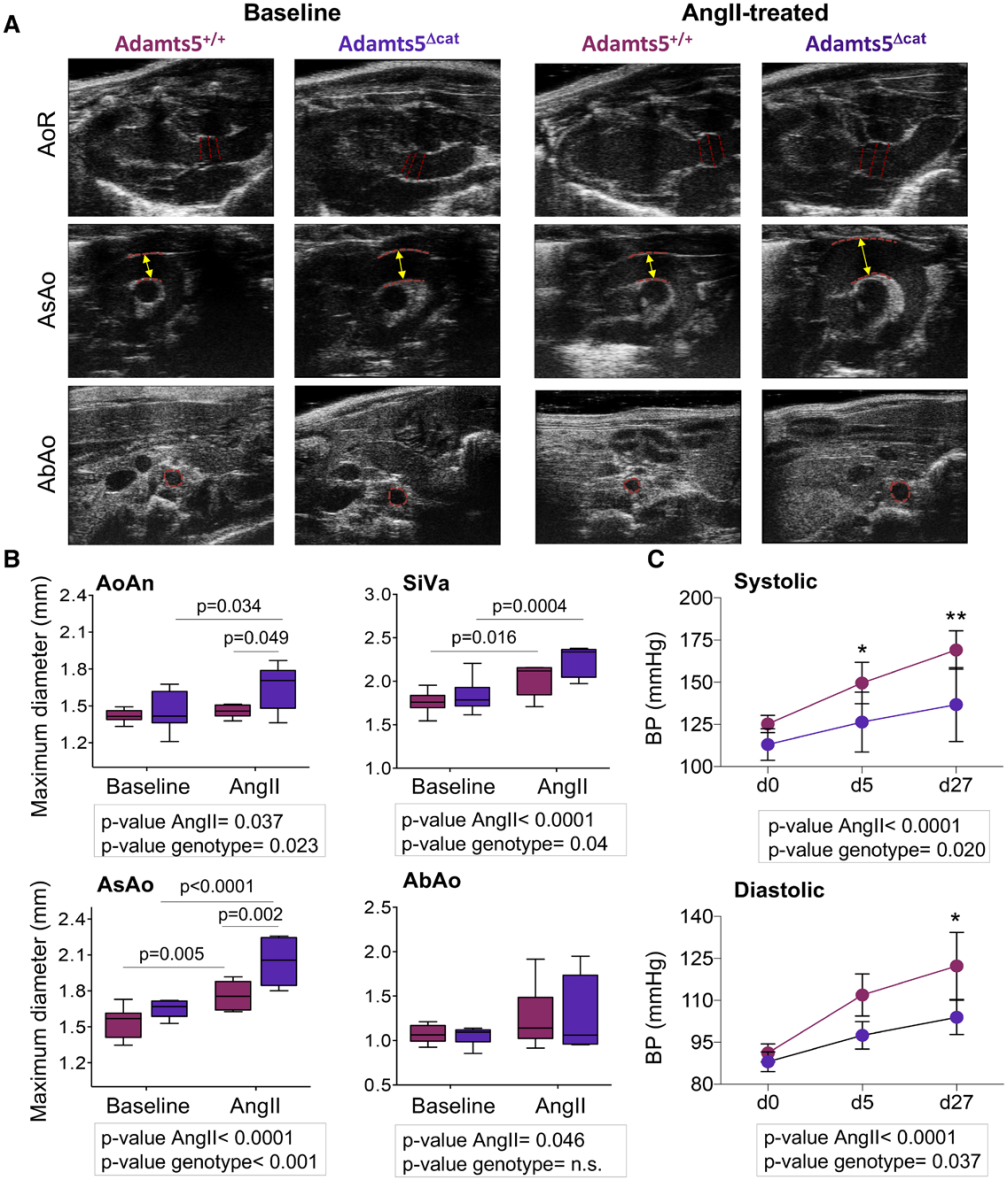
Aortic dilatation in mice lacking the region of the catalytic domain of a disintegrin and metalloproteinase with thrombospondin motifs-5 (Adamts5^Δcat^ mice). **A**, Representative ultrasound images. Red dashed lines mark areas considered for aortic diameter measurements. **B**, Maximal diameters of the aortic ring (AoR; aortic annulus [AoAn] and sinuses of Valsalva [SiVa]), ascending aorta (AsAo), and abdominal aorta (AbAo) from AngII (angiotensin II)-treated (Adamts5^+/+^, n=6 and Adamts5^Δcat^, n=5) and untreated (Adamts5^+/+^, n=9 and Adamts5^Δcat^, n=9) mice. Adamts5^+/+^ and Adamts5^Δcat^ mice are represented using burgundy and purple color, respectively. **C**, Systolic and diastolic blood pressure (BP) measurements at day 0, 5, and after 27 days of AngII infusion in Adamts5^+/+^ (n=6) and Adamts5^Δcat^ mice (n=4). Data points are mean±SD; *P* values were derived from a 2-way ANOVA (aortic diameter) and 1-way ANOVA (blood pressure) with Šidák correction. *P<0.05, **P<0.01.

Loss of ADAMTS-1 has recently been implicated in TAA.^3^ Thus, we assessed aortic dilatation in mice lacking the region encoding for the catalytic domain of Adamts5 (Adamts5^Δcat^).^21^ Hypertension was induced in Adamts5^+/+^ and Adamts5^Δcat^ mice by AngII infusion for 4 weeks (Figure I in the online-only Data Supplement). AngII treatment was associated with increased aortic dilatation in Adamts5^Δcat^ mice (Figure 2A and 2B); significant differences compared with Adamts5^+/+^ were observed both in the aortic root, in particular in the aortic annulus, and the AsAo. Notably, the increased aortic dilatation in Adamts5^Δcat^ mice occurred despite an attenuated rise in systolic and diastolic blood pressure after 27 days of AngII treatment (Figure 2C). The aortic phenotype and the blood pressure changes in Adamts5^Δcat^ mice bear a strong resemblance to the one recently described in mice with Adamts1 haploinsufficiency.^3^

### Vascular Substrates of ADAMTS-1 and ADAMTS-5

**Figure 3.**
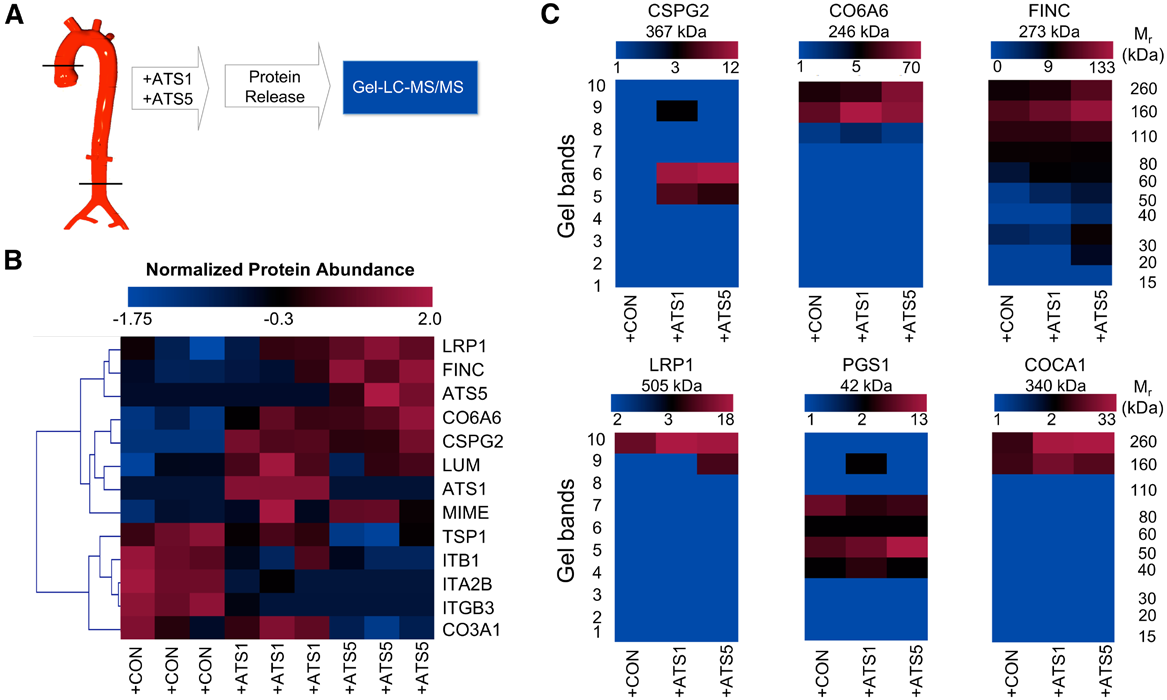
Proteomics to identify vascular targets of ADAMTS (a disintegrin and metalloproteinase with thrombospondin motifs)-1 and -5. **A**, Murine aortas were subjected to overnight incubation with either 50 pmol/L ADAMTS-1 (+ATS1) or 50 pmol/L ADAMTS-5 (+ATS5) at 37°C. Aortic explants incubated in buffer served as control (+CON). **B**, Unsupervised hierarchical clustering of the differentially released ECM (extracellular matrix) proteins (55 proteins were tested by Kruskal–Willis test, and 13 were found differentially regulated; *P*<0.05) in the supernatants of mouse aortas as identified by proteomics (n=3). The blue–red heat map represents normalized spectral counts highlighting differences in proteins released from aortic explants. **C**, Spectral evidence for fragmentation. The color-coded heat map represents the total number of identified spectra in each gel band and visualizes the fragmentation of ECM proteins. Color scales for heat maps represent maximum and minimum number of spectra identified in each group. COCA1 indicates collagen type XII α1 chain; CO6A6, collagen type VI α6 chain; CSPG2, versican; FINC, fibronectin; LC-MS/MS, liquid chromatography-tandem mass spectrometry; LRP1, low-density lipoprotein receptor-related protein 1; and PGS1, biglycan.

To identify vascular substrates of ADAMTS-1 and ADAMTS-5, murine aortas were subjected to overnight incubation with either ADAMTS-1 or ADAMTS-5 (50 pmol L^−1^ mg^−1^ tissue at 37°C; Figure 3A). Aortas incubated in buffer only served as control. The proteins released into the digestion buffer were separated by SDS-PAGE, subjected to in-gel tryptic digestion, and analyzed by liquid chromatography-tandem mass spectrometry. Among the identified extracellular proteins, proteins with differential release on digestion by ADAMTS-1 and -5 include known substrates such as CSPG2 (versican; Figure 3B; Table I in the online-only Data Supplement). Detailed examination of the gel-liquid chromatography-tandem mass spectrometry data revealed the presence of proteolytic products (Figure 3C), that is, for CSPG2, CO6A6 (collagen type VI α-6), FINC (fibronectin), and LRP1. However, proteolytic cleavage may occur without differential release into the digestion buffer, that is, a higher number of MS/MS spectra for PGS1 (biglycan) and COCA1 (collagen type XII α-1) were identified in gel segments below the expected molecular weight of the full-length protein, indicative of proteolysis. Again, this includes known substrates of ADAMTS-5, such as PGS1.^22^

### Proteomics Analysis of Aortas From AngII-Treated Wild-Type and Adamts5^Δcat^ Mice

**Figure 4.**
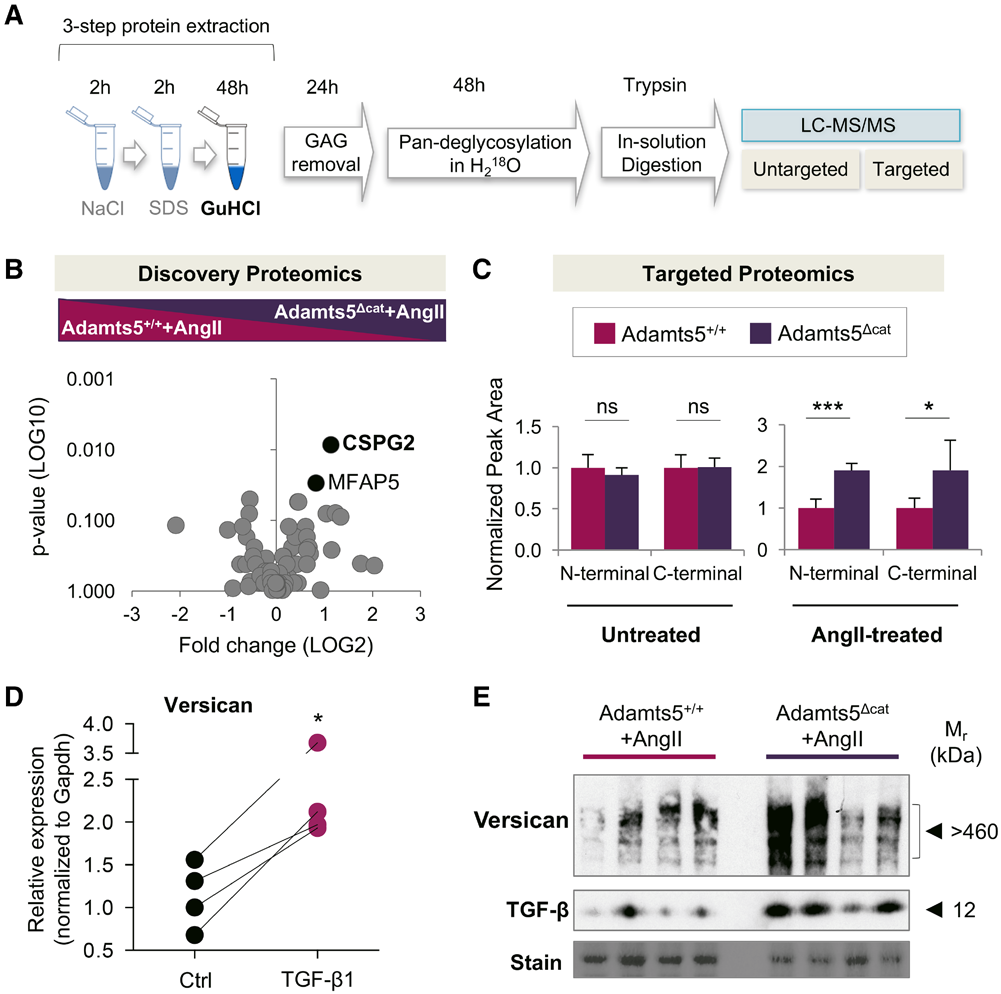
ECM (extracellular matrix) proteomics of AngII (angiotensin II)-treated murine aortas. **A**, ECM proteomics workflow. **B**, ECM extracts of the entire aorta from AngII-treated Adamts5^+/+^ (a disintegrin and metalloproteinase with thrombospondin motifs; n=6) and Adamts5^Δcat^ (n=5) mice were analyzed using discovery proteomics. Black dots represent proteins differentially regulated between the groups (*P*<0.05). Statistical significance was calculated using Mann–Whitney test (76 ECM and ECM-associated proteins were tested, and 2 were found differentially regulated). **C**, Two different proteotypic peptides (ie, N-terminal and C-terminal) were chosen to validate levels of versican in aortic tissue using targeted proteomics. Peak area values of versican in Adamts5^+/+^ served as reference for N-terminal and C-terminal peptides. Statistical significance was calculated using unpaired Student *t* test. **D**, Internal mammary artery smooth muscle cells (SMCs) from 4 donors were incubated with TGF (transforming growth factor)-β1. Gene expression of versican was assessed by quantitative PCR. Gapdh was used as internal control. Statistical significance was calculated using unpaired Student *t* test. **E**, Immunoblots performed on total protein lysates reveal an increase in full-length versican and TGF-β in aortas of AngII-treated Adamts5^Δcat^ mice (n=4 per group). CSPG2 denotes versican; GAG, glycosaminoglycan; GuHCl, guanidine hydrochloride; LC-MS/MS, liquid chromatography-tandem mass spectrometry; MFAP5, microfibrillar-associated protein 5; and n.s., not significant.

To analyze the changes in ECM composition on loss of ADAMTS-5 activity, aortas from AngII-treated Adamts5^+/+^ and Adamts5^Δcat^ mice were subjected to a 3-step sequential extraction procedure: pretreatment with 0.5 mol/L NaCl followed by decellularization with 0.08% SDS and a final incubation step with 4 mol/L GuHCl to solubilize the ECM (Figure 4A). Using a discovery proteomics approach, along with MFAP5 (microfibrillar-associated protein 5), CSPG2 was returned as more abundant in aortas of AngII-treated Adamts5^Δcat^ mice (Figure 4B; Table II in the online-only Data Supplement). Subsequent targeted proteomics analysis using both N-terminal and C-terminal proteotypic peptides corroborated that versican was elevated in AngII-treated Adamts5^Δcat^ mice (Figure 4C; Table III in the online-only Data Supplement). TGF-β1 is known to enhance versican expression.^23^ Human arterial SMCs stimulated with TGF-β1 showed a rise in versican transcripts (Figure 4D). Notably, higher levels of versican were associated with increased protein abundance of TGF-β in aortas of AngII-treated Adamts5^Δcat^ mice (Figure 4E; Figure II in the online-only Data Supplement). Gene expression levels of *Tgfb1 and 2* and their receptors *Tgfbr1-3* were significantly higher in Adamts5^Δcat^ mice at baseline (Figure III in the online-only Data Supplement). Interestingly, *Adamts1* transcripts were markedly elevated in Adamts5^Δcat^ mice, suggesting a compensatory increase of ADAMTS-1 on loss of ADAMTS-5 activity.

### Versican Cleavage in Aortas of Adamts5^Δcat^ Mice

**Figure 5.**
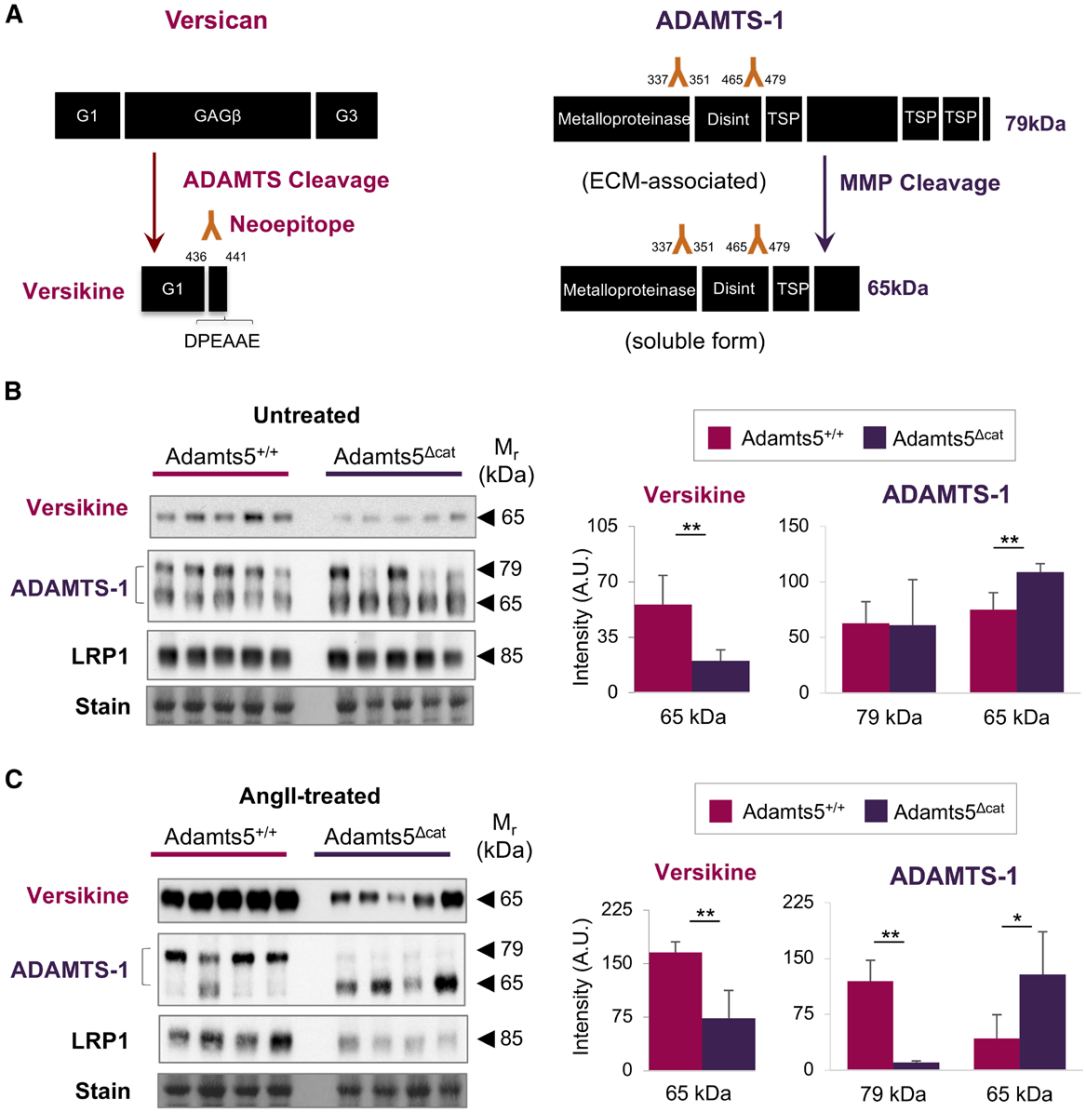
ADAMTS (a disintegrin and metalloproteinase with thrombospondin motifs)-1 cannot compensate for the lack of ADAMTS-5 activity. **A**, **Left**, Schematic representation of ADAMTS-mediated versican processing. The signature cleavage site for ADAMTS activity gives rise to an N-terminal versican fragment (versikine) and is recognized by a neoepitope antibody to the DPEAAE^441^ amino acid sequence. **Right**, Schematic representation of ADAMTS-1 processing. Processing by MMPs (matrix metalloproteinases) gives rise to a truncated, soluble form of ADAMTS-1 (65 kDa). **B**, **C**, Immunoblots using neoepitope antibodies to versikine were performed on aortic ECM (extracellular matrix) extracts from untreated and AngII (angiotensin II)-treated mice (n=5 per group). Immunoblots for ADAMTS-1 and LRP1 (low-density lipoprotein-related protein 1) were performed on total protein lysates of untreated and AngII-treated mice (n=5 and n=4 per group, respectively). Densitometry for the versican fragment (versikine) and ADAMTS-1. Bars represent mean±SD. Statistical significance was calculated using unpaired Student *t* tests. *P<0.05, **P<0.01. A.U. indicates arbitrary unit; and TSP, trombospondin domain.

Versican was the most differentially regulated ECM protein in aortas from Adamts5^Δcat^ mice treated with AngII (Figure 4B). It was also returned as one of the major substrates of ADAMTS-1 and -5 in the analysis of aortic explants (Figure 3B). Therefore, we focused on ADAMTS-mediated versican processing. ADAMTS activity results in the release of a specific versican fragment ending with the DPEAAE^441^ sequence,^11,24^ named versikine.^25^ This signature cleavage site of ADAMTS activity can be detected using a neoepitope antibody (Figure 5A). As expected, aortas from Adamts5^Δcat^ mice displayed reduced versikine; the 65 kDa band is representative of versikine (Figure 5B). Even after AngII treatment, less versikine was observed in Adamts5^Δcat^ mice. This highlights the dependence on ADAMTS-5 activity for versicanolysis in AngII-induced aortic remodeling (Figure 5C).

### Compensatory Increase of ADAMTS-1 in Aortas of Adamts5^Δcat^ Mice

Because of different processing, ADAMTS-1 can be found in 2 forms, a larger, cell layer and ECM-associated version, and a smaller, soluble version (Figure 5A).^26^ Immunoblotting for ADAMTS-1 performed on total aortic tissue lysates revealed that the loss of ADAMTS-5 activity was accompanied by an increase in the smaller, soluble form of ADAMTS-1 (65 kDa). This was exacerbated after AngII treatment. In contrast, the larger, ECM-associated ADAMTS-1 form (79 kDa) was detected almost exclusively in the wild-type group. The increase in soluble ADAMTS-1 suggests a cross-talk between members of the ADAMTS protease family; however, this compensatory upregulation did not result in the complete rescue of ADAMTS-mediated versican cleavage (Figure 5C).

### Reduced LRP1 in AngII-Treated Aortas of Adamts5^Δcat^ Mice

**Figure 6.**
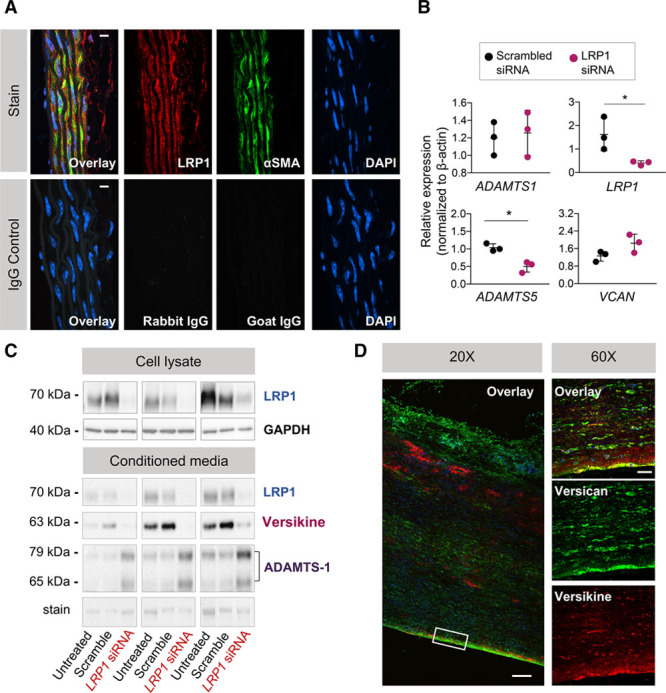
Effects of LRP1 (low-density lipoprotein-related protein 1) and detection of versikine in human ascending aortic aneurysms. **A**, LRP1 localization in the ascending aorta of mice lacking the catalytic domain of a disintegrin and metalloproteinase with thrombospondin motifs 5 (Adamts5^Δcat^ mice). Colocalization of LRP1 (Alexa 647, displayed in red) and αSMA (alpha smooth muscle actin; Alexa 568, displayed in green) in the mouse aorta as visualized by immunofluorescence. Elastin fibers are in white (autofluorescence captured with 488 nm laser excitation). Control sections stained with isotope IgGs. Magnification ×60, scale bars=10 μm. **B**, Relative gene expression of *ADAMTS1*, *LRP1*, *ADAMTS5*, and *VCAN* in human aortic smooth muscle cells (SMCs) treated for 72 h with *LRP1* siRNA (n=3). β-actin was used as reference gene. Data points are mean±SD. Statistical significance was calculated using paired Student *t* tests. *P<0.05. **C**, Immunoblotting performed on cell lysate and conditioned media after transfection (n=3). Silencing LRP1 results in a decrease in versikine and an increase in ADAMTS-1 in the conditioned media. **D**, Localization of versican (Alexa568, displayed in green) and versikine (Alexa 647, displayed in red) in human ascending aortic aneurysms as visualized by immunofluorescence. Magnification ×20, scale bar=200 μm and ×60, scale bar=20 μm.

To further investigate the molecular mechanisms associated with the loss of ADAMTS-5 activity, we analyzed LRP1, a receptor implicated in the endocytosis of ADAMTS proteases. LRP1 was identified as a vascular target of ADAMTS-1 and -5 (Figure 3). LRP1 was profoundly reduced in aortas of AngII-treated Adamts5^Δcat^ mice (Figure 5C; Figure IV in the online-only Data Supplement). LRP1 staining was mainly observed in SMCs of mouse aortas (Figure 6A). Next, we silenced *LRP1* expression in primary human aortic SMCs. Silencing LRP1 did not affect *ADAMTS1* gene expression but markedly reduced expression of *ADAMTS5* (Figure 6B). Consequently, versikine was barely detectable in the conditioned media of SMCs transfected with an siRNA to *LRP1* (Figure 6C; Figure V in the online-only Data Supplement). Moreover, in agreement with our results in AngII-treated Adamts5^Δcat^ mice, we observed a concomitant increase in ADAMTS-1 on repression of LRP1. Thus, these in vitro experiments complement our in vivo findings and establish a link between LRP1 and ADAMTS-5–mediated versican cleavage.

### Versican Cleavage in Human Aortic Aneurysms

Although differences in versican abundance have been reported in human abdominal aortic aneurysms,^27^ versikine has not been localized yet in human AsAo aneurysms. Immunofluorescence staining was performed for versican and versikine. Versican staining was seen throughout the aortic wall, whereas versikine was localized predominantly in the outer media and the subintimal layer of the AsAo aneurysm (Figure 6D). These data confirm the presence of ADAMTS-specific versican cleavage in human ascending TAA tissue.

## Discussion

We have characterized regional differences in ADAMTS expression along the murine aorta and used proteomics to evaluate the effect of loss of ADAMTS-5 activity on aortic ECM remodeling. We demonstrated that deficiency of ADAMTS-5 affects ADAMTS-1 gene expression, protein abundance and processing and resulted in aortic dilatation similar to the phenotype recently described in Adamts1-haploinsufficient mice.^3^

### ADAMTS Proteases in Murine Aortas

We have previously demonstrated that ADAMTS-5 is the most potent protease associated with versican cleavage in the murine aorta.^11^ Loss of ADAMTS-5 also results in the accumulation of aggrecan.^12^ To investigate the contribution of ADAMTS-5 in ECM remodeling during aortic aneurysm formation, a mouse model lacking ADAMTS-5 activity was used. Administration of AngII for 4 weeks exacerbated aortic dilatation in Adamts5^Δcat^ mice, with a significantly greater diameter in the AsAo where *Adamts5* expression is the highest in Adamts5^+/+^ mice. Moreover, Adamts5^Δcat^ mice showed an attenuated rise in blood pressure in response to AngII stimulation compared to Adamts5^+/+^ mice. Interestingly, a recent study reported a similar effect on blood pressure in Adamts1-haploinsufficient mice.^3^ Also other ADAMTS proteases have been implicated in the vasculature and the regulation of blood pressure.^28^

### Proteomics to Identify Vascular Substrates

In the current study, we used proteomics for the analysis of aortic ECM changes associated with loss of ADAMTS-5 activity. By using proteomics, we found that versican was more abundant in AngII-treated Adamts5^Δcat^ mice. Versican is one of the most abundant large aggregating proteoglycans in the aorta.^16^ Tissue accumulation of versican has been linked to the loss of fibrous ECM organization.^29^ Because we failed to detect changes in versican gene expression in Adamts5^Δcat^ mice (data not shown), differences in versican protein abundance are most probably related to reduced degradation rather than protein synthesis. Versican is a well-known substrate of ADAMTS proteases.^30^ Versican processing resulting in the release of versikine was also observed after incubation of human aortic tissues with either ADAMTS-1 or ADAMTS-5.^11^ The absence of the catalytic activity of ADAMTS-5 seems to result in the build-up of unprocessed versican in the aortic wall, as previously observed in valve tissue of Adamts5^Δcat^ mice.^13^

### ADAMTS-Mediated Versicanolysis

Versican can be cleaved by different proteases including MMPs,^31^ plasmin,^32^ and several members of the ADAMTS family. The latter harbor sequence motifs specific to large aggregating proteoglycans, including versican. The neoepitope DPEAAE at position 441 of versican is generated after ADAMTS cleavage.^25^ The neoepitope antibody recognizes only the cleaved product of versican, corresponding to the N-terminus of the proteoglycan.^25^ This fragment was reduced in Adamts5^Δcat^ mice, which is in agreement with our previous report.^11^ As demonstrated in this study, this effect is exacerbated after AngII infusion; chronic infusion of AngII enhanced the release of versikine in murine aortas. In AngII-treated Adamts5^Δcat^ mice, immunoblotting revealed a marked reduction of the ADAMTS-specific DPEAAE neoepitope demonstrating the importance of ADAMTS-5 for versican cleavage. Versikine has been suggested to stimulate SMC migration and induce cell apoptosis during development.^33,34^ Little residual ADAMTS activity was detectable in aortas of AngII-treated Adamts5^Δcat^ mice. The latter is most likely attributable to ADAMTS-1, which was increased in Adamts5^Δcat^ mice. ADAMTS-1 is known to be processed into 2 active forms: (1) an ECM-associated and (2) a soluble form, generated after MMP-mediated cleavage of the thrombospondin domain at the C-terminus. The loss of ADAMTS-5 activity led to a rise in the soluble form of ADAMTS-1, and this effect was exacerbated after AngII treatment. The removal of the last thrombospondin domain renders ADAMTS-1 more similar to ADAMTS-5, suggesting that the release of soluble ADAMTS-1 could be a compensatory response to the loss of ADAMTS-5. ADAMTS-1, however, cannot sufficiently compensate for the lack of ADAMTS-5 activity, at least with regards to versican cleavage.

### Effects on TGF-β

The increase in total versican levels was associated with a concomitant upregulation of TGF-β in Adamts5^Δcat^ mice after AngII treatment. TGF-β is known to induce the expression of different proteoglycans, including versican.^23^ In line with this finding, stimulation of arterial SMCs with TGF-β1 led to an increase in versican expression. Interestingly, it has also been shown that the latent form of TGF-β can be associated and subsequently activated by the C-terminal region of ADAMTS-1, thereby enhancing the inflammatory response in liver fibrosis.^35^ In agreement with these results, we found a direct correlation between ADAMTS-1 and TGF-β regulation in ADAMTS-5^Δcat^ mice on AngII treatment. Increased levels of versican, TGF-β, and ADAMTS-1 were accompanied by an increased aortic dilatation in these mice after AngII treatment. Dysregulation of TGF-β has been linked to aneurysm development.^36,37^ However, the mechanisms of TGF-β–mediated aneurysm formation have not been completely elucidated yet. The present study highlights an effect of ADAMTS activity on TGF-β bioavailability.

### Regulation by LRP1

LRP1 has previously been implicated in aortic aneurysm formation in humans and mice^14,38,39^; however, the exact mechanisms are unclear. LRP1 is involved in different cellular processes, such as lipid homeostasis, signal transduction, and endocytosis.^40,41^ LRP1 also acts as a protease sink responsible for cellular uptake of ADAMTS-5 from the extracellular space.^42^ Moreover, LRP1 can bind TGF-β1 and TGF-β2 and thereby inhibit cell proliferation and regulate vascular remodeling, respectively.^43^ After AngII treatment, LRP1 was less abundant in aortas of Adamts5^Δcat^ mice. This was accompanied by an increase in the short soluble form of ADAMTS-1 as a possible mechanism to compensate for the lack of ADAMTS-5. This coordinated regulation is consistent with a feedback mechanism between ADAMTS-5 and LRP1. A compensatory downregulation of LRP1 in AngII-treated Adamts5^Δcat^ mice could increase the extracellular availability of other ADAMTS proteases. Silencing LRP1 in human aortic SMCs reduced *ADAMTS5* expression and resulted in a loss of ADAMTS-mediated versican cleavage. Again, this was accompanied by an increase of ADAMTS-1 in the conditioned media. These findings expand on a previous report that the deletion of *Lrp1* in SMCs augmented AsAo dilatation in AngII-treated mice.^14^

### Limitations of the Study

A mouse model of AngII infusion was used to study the effects of ADAMTS-5 on aortic dilatation, which may not recapitulate all aspects of human aneurysmal disease, in particular with regards to hemodynamics and mechanotransduction.^44,45^ Also, the relative importance of different members of the ADAMTS family may differ between species. In human aneurysmal disease, previous studies have shown increased protein and transcript levels of ADAMTS-1 and -4 in TAA.^10^ Consistent with our findings, a recent study reported decreased *ADAMTS5* expression and increased deposition of proteoglycans, such as aggrecan and versican in human TAA.^8^ Finally, changes in ADAMTS activity are likely to be associated with the proteolysis of other proteins apart from versican.

### Conclusions

At present, the clinical management of aneurysms is hampered by our limited knowledge about the cause and pathogenic mechanisms involved in the disease.^46^ Evidence is emerging that ECM processing by members of the ADAMTS protease family could play an important role in the progression of aortic dilatation.^3^ The present study takes advantage of proteomics to define vascular substrates of ADAMTS proteases. LRP1, which has been involved in aneurysm pathology in both humans and mice, appears to be linked to ADAMTS-5 and ADAMTS-5–mediated versican cleavage. In mice lacking ADAMTS-5 activity, AngII infusion results in decreased versicanolysis, an increase in full-length versican and TGF-β, but reduced LRP1 compared with wild-type controls. Mice lacking ADAMTS-5 activity also showed increased aortic dilatation in response to AngII infusion despite a lower blood pressure. A compensatory rise in ADAMTS-1 could not prevent aortic dilatation in Adamts5^Δcat^ mice, suggesting a greater contribution of ADAMTS-5 in this context. Further studies are needed to explore the ADAMTS protease family as a therapeutic target to reduce or halt dilatation of the aorta resulting in dissection.

## Acknowledgments

All authors have read and approved the article. We thank Dr Karen E Porter (Leeds, United Kingdom) for providing extracts from human arterial smooth muscle cells.

## Sources of Funding

M. Mayr is a British Heart Foundation (BHF) Chair Holder (CH/16/3/32406) with BHF programme (RG/16/14/32397) and project grant support (PG/17/48/32956). The research was supported by National Institute of Health Research (NIHR) Biomedical Research Centre based at Guy’s and St Thomas’ NHS (National Health Service) Foundation Trust and King’s College London in partnership with King’s College Hospital. The study was also supported by St. George’s Hospital Charitable Foundation, University of London, and by an excellent initiative (Competence Centers for Excellent Technologies [COMET]) of the FFG (Austrian Research Promotion Agency): Research Center of Excellence in Vascular Ageing-Tyrol, VASCage (K-Project No. 843536) funded by BMVIT (Federal Ministry for Transport, Innovation and Technology), BMWFW (Federal Ministry of Science, Research and Economy), the Wirtschaftsagentur Wien, and Standortagentur Tirol.

## Disclosures

None.

## Highlights

- Expression of ADAMTS-5 (a disintegrin and metalloproteinase with thrombospondin motifs-5) and the large aggregating proteoglycans is higher in the aortic arch compared with the abdominal aorta.
- AngII (angiotensin II) treatment in Adamts5^Δcat^ mice is associated with increased dilatation of the ascending aorta.
- Loss of ADAMTS-5 activity induces an accumulation of versican on AngII treatment.
- This is accompanied by an increase in TGF-β (transforming growth factor-β) levels and a compensatory upregulation of soluble ADAMTS-1.
- Silencing of LRP1 (low-density lipoprotein-related protein 1) in human aortic smooth muscle cells reduces ADAMTS-5 expression, attenuates ADAMTS-mediated versican cleavage, and increases ADAMTS-1.
